# Therapie der Hyperglykämie bei erwachsenen, kritisch kranken PatientInnen (Update 2023)

**DOI:** 10.1007/s00508-023-02173-9

**Published:** 2023-04-20

**Authors:** M. Clodi, M. Resl, H. Abrahamian, B. Föger, R. Weitgasser

**Affiliations:** 1 Abteilung für Innere Medizin, Konventhospital der Barmherzigen Brüder Linz, Linz, Österreich; 2 ICMR – Institute for Cardiovascular and Metabolic Research, Johannes Kepler Universität Linz (JKU Linz), Altenberger Straße 69, 4040 Linz, Österreich; 3 Privates Institut für Medizin&NLP, wissenschaftliches Institut gemäß BundesstatistikG 2008 ÖNACE-CODE: 72.19-0, Wien, Österreich; 4 Abteilung für Innere Medizin, Kreiskrankenhaus Pfarrkirchen, Pfarrkirchen, Deutschland; 5 Kompetenzzentrum Diabetes, Privatklinik Wehrle-Diakonissen, Salzburg, Österreich; 6 Paracelsus Medizinische Privatuniversität Salzburg, Salzburg, Österreich

**Keywords:** Hyperglykämie, Stresshyperglykämie, Kritisch Kranke, Blutzuckertherapie, Hyperglycemia, Stresshyperglycemia, Critically ill, Glucose management

## Abstract

Bei kritisch kranken PatientInnen kommt es häufig zum Auftreten einer Hyperglykämie welche eindeutig mit einer gesteigerten Mortalitätsrate assoziiert ist. Der aktuell verfügbaren Datenlage entsprechend sollte eine intravenöse Insulintherapie bei Blutzuckerwerten > 180 mg/dl begonnen werden und ein Zielbereich zwischen meist 140–180 mg/dl angestrebt werden.

## Abstract (en)

In critical illness hyperglycemia is associated with increased mortality. Based on the currently available evidence, an intravenous insulin therapy should be initiated when blood glucose is above 180 mg/dl. After initiation of insulin therapy blood glucose should be maintained between 140 and 180 mg/dl.

Diese Leitlinie wurde für die Therapie von Erwachsenen, kritisch kranken PatientInnen erstellt. Seit dem Jahr 2019 haben sich basierend auf der nun aktuellen Datenlage die prinzipiellen Empfehlungen nicht wesentlich verändert.

Bei akut kranken PatientInnen kommt es häufig zum Auftreten einer Hyperglykämie, welche die Mortalitätsrate unabhängig von einem vorbekannten Diabetes mellitus erhöht. Generell bewirkt die Hyperglykämie eine Vielzahl an Komplikationen wie beispielsweise eine erhöhte kardiovaskuläre Ereignisrate oder eine erhöhte Inzidenz von Thrombosen. Weiters wirkt die Hyperglykämie proinflammatorisch und verzögert die Wundheilung [1].

Die Dekompensation eines bereits vorhandenen Diabetes ist die häufigste Ursache für das Auftreten von Hyperglykämien bei kritisch kranken Patienten. Generell werden 3 unterschiedliche Ätiologien in der Literatur beschrieben.

- Patienten mit bereits bekanntem Diabetes.
- Patienten mit noch unerkanntem Diabetes.
- krankheitsassoziierte Hyperglykämie, welche nach Entlassung nicht mehr nachweisbar ist.

Eine Unterscheidung der beschriebenen Ätiologien kann mit Hilfe der Anamnese, bzw. des HbA1c Wertes erfolgen, wobei bei einem HbA1c-Wert > 6,5 % mit hoher Sicherheit von einem primär unerkanntem Diabetes auszugehen ist [2].

Aus heutiger Sicht ist nicht eindeutig geklärt, ob die Hyperglykämie während eines Aufenthaltes auf der Intensivstation immer als Prädiabetes zu werten ist, da nur ein geringer Anteil dieser Patienten nach Entlassung tatsächlich einen manifesten Diabetes mellitus entwickelt. Dennoch ist nach vollständiger Genesung eine formelle Diagnostik erforderlich.

In Analogie zu den aktuellen Leitlinien der American Diabetes Association sollte eine Insulintherapie bei kritisch kranken Patienten ab Blutglukosewerten ≥ 180 mg/dl initiiert werden. Nach Beginn der Insulintherapie liegt der Glukose-Zielbereich zwischen 140 mg/dl und 180 mg/dl. Basierend auf der heute verfügbaren Datenlage gilt die kontinuierliche Insulininfusion nach wie vor als Mittel der Wahl für die optimale Blutzuckertherapie [2, 3]. Für die Auswahl des Insulins (Human- oder Analoginsulin) gibt es derzeit keine konklusiven Daten, welche vorteilhafte Effekte einer Therapie mit Analoginsulinen im Vergleich zu Humaninsulinen belegen.

Die wissenschaftliche Evidenz für diese Empfehlungen, wurde durch zahlreiche große Studien und Metaanalysen geschaffen [4–6]. Dennoch wird die Qualität der Daten darunter auch die der NICE-Sugar Studie und der „Van den Berghe“ Studien in einer Empfehlung des American College of Physicians als mittelmäßig eingestuft [5]. Dieser Empfehlung zu Folge existiert bis heute keine einzige Arbeit, deren Evidenz als hochwertig zu beurteilen wäre.

Im Rahmen der NICE-Sugar Studie (6104 Patienten), welche zu den aktuellsten, und auch größten Studien zählt, wurden die Effekte unterschiedlicher Blutzuckerzielwerte auf Mortalität untersucht. Am Beginn der Studie wurden die Patienten in eine intensivierte Therapiegruppe (81–108 mg/dl), und eine Standard-Therapiegruppe (144–180 mg/dl) randomisiert. Die Patienten der intensivierten Therapiegruppe hatten verglichen mit den Patienten der Standardgruppe eine signifikant höhere 90-Tage Mortalitätsrate (27,5 % vs. 24,9 %). Dieser Effekt war unabhängig von der Art der Intensivstation (Intern oder Chirurgisch). Die Frequenz schwerer Hypoglykämien war in der intensivierten Therapiegruppe mit 6,8 % signifikant höher als in der Vergleichsgruppe 0,5 %. Die genauen Ursachen für die gesteigerte Mortalität in der intensivierten Therapiegruppe sind allerdings unklar [7].

Entgegen dieser Ergebnisse konnte eine Studie von Van den Berghe und Kollegen eine Reduktion der Mortalität durch eine intensivierte Blutzuckertherapie bei Patienten, einer chirurgischen Intensivstation darstellen [8]. In dieser Studie wurden Blutzuckerwerte zwischen 80 und 110 mg/dl angestrebt.

Eine Metaanalyse von Griesdale und Kollegen, welche die Daten aller wichtigen Studien beinhaltet, konnte eine relative Mortalitätsreduktion von 7 % bewirkt durch eine intensivierte Insulintherapie zeigen [4].

Die meisten Studien haben belegt, dass eine intensivierte Therapie das Risiko für schwere Hypoglykämien deutlich erhöht.

Entsprechend dieser Metaanalyse und den Daten von Van den Berghe profitieren gerade Patienten chirurgischer Intensivstationen am meisten von einer strikten Blutglukosekontrolle (Relatives Risiko 0,63).

Bei Patienten internistischer Intensivstationen lag das relative Risiko bei 1,0. Somit konnten weder vorteilhafte noch negative Effekte dargestellt werden.

In jedem Fall sollte ein Blutzuckerwert von 110 mg/dl nicht unterschritten werden [2].

Dies gilt besonders deshalb, weil gerade bei intensivpflichtigen PatientInnen zahlreiche Risikofaktoren für das Auftreten von Hypoglykämien vorliegen. Zu diesen Faktoren gehören ein reduzierter Ernährungszustand, Komorbiditäten wie Herzinsuffizienz, Leber- und Niereninsuffizienz, Malignome, Infektionen oder Sepsis. Weiters können Hypoglykämien durch plötzliche Reduktion von hohen Glukokortikoidtherapiedosen, Übelkeit und Erbrechen und Modifikationen der enteralen und parenteralen Therapie getriggert werden (z. B. Pausierung der kontinuierlichen, enteralen Ernährung, bzw. vor Interventionen Gastroskopie, Extubationsversuch). Hypoglykämien sollten bereits vor ihrem Auftreten antizipiert werden, und eine Modifikation der Therapie noch vor Beginn der Episode erfolgen.

Regelmäßige Blutglukosekontrollen sollten bei oraler Ernährung alle 4–6 h erfolgen. Wird ein Patient mittels kontinuierlicher Insulinsubstitution behandelt, sollte die Blutglukose je nach Blutzuckerspiegel, Insulindosis und Stabilität des Verlaufs alle 30 min bis 3 h gemessen werden. Weiterhin liegen für Continuous Glucose Monitoring Systeme (CGM) und Flash Glucose Monitoring Systeme (FGM) keine Zulassungen für die Anwendung bei kritisch kranken PatientInnen vor. Dennoch existieren bereits eindrückliche Daten, welche die Reduktion von schweren Hypoglykämien belegen [9].

Zur Vermeidung von Hyperglykämien nach Beendigung der intravenösen Insulintherapie wird die Anwendung eines standardisierten Transitionsprotokolles empfohlen. Falls erforderlich soll ein entsprechendes Basalinsulin bereits 2–4 h vor der Beendigung der intravenösen Insulingabe appliziert werden.

## Empfehlungen

- Initiierung einer kontinuierlichen, intravenösen Insulintherapie bei Blutzuckerwerten um 180 mg/dl (venöses Plasma) (Evidenzlevel A).
- Unter der Insulintherapie sollten Blutglukosewerte zwischen 140–180 mg/dl angestrebt werden (Evidenzlevel A).
- In bestimmten Kollektiven (chirurgische Intensivstation, elektive Eingriffe) sollten Blutzuckerwerte zwischen 110–140 mg/dl angestrebt werden (Evidenzlevel C).
- Die intravenöse Insulintherapie gilt als Mittel der Wahl bei kritisch kranken PatientInnen.
- Regelmäßige Kontrollen der Blutzuckerwerte; besonders bei Patienten mit hohem Risiko für Hyperglykämie (Evidenzlevel B)
  - Parenterale Ernährung
  - Glukokortikoidtherapie
  - Immunsuppressive Medikamente
  - Octreotid
- HbA_1c_-Bestimmung (bei Aufnahme)
- Formelle Glukosediagnostik nach Transfer oder Entlassung
- Rasches, effektives Management von Hypoglykämien
- Weiterhin liegen für Continuous Glucose Monitoring Systeme (CGM) und Flash Glucose Monitoring Systeme (FGM) keine Zulassungen für die Anwendung bei kritisch kranken Patienten vor.
